# Beyond borders: investigating microbiome interactivity and diversity for advanced biocontrol technologies

**DOI:** 10.1111/1751-7915.12235

**Published:** 2015-01-27

**Authors:** Gabriele Berg

**Affiliations:** Institute of Environmental Biotechnology, Graz University of Technology & ACIB Austrian Centre of Industrial BiotechnologyGraz, Austria

## Microbial biotechnology, crystal ball

Recent, primarily technology-based advances in microbial ecology have opened an immense treasure chest of microbial diversity that has been observed in the vast majority of all investigated habitats. Additionally, habitats which were assumed to be sterile for a long time are now known to be colonized by diverse microorganisms; e.g. within the placenta and stomach of humans or reproductive organs of plants. Through the implementation of new techniques, deeper insights into the structure of microbiomes mainly by amplicon sequencing and microscopy have been gained. Now the current focus of research is on analysis of their function implementing meta-(genomic/epigenomic/transcriptomic/proteomic) techniques. The management of the accumulated metadata and the gleaning of new information is the challenge we are facing, but that challenge will be solved in the near future (Jansson, 2013). By looking into the proverbial crystal ball, my intention is to highlight two scientific aspects in particular, which I believe have been overlooked in current approaches to ecological research: (i) the interactivity of microbiomes and (ii) the interplay between microorganisms derived from different kingdoms – in particular archaea, bacteria and fungi. Both aspects could inspire the future course of biotechnology.

In recent decades, deeper insight into many aquatic and terrestrial microbial communities was gained using omics approaches. Although this is now possible, considerable time must be invested in the future in order to assemble all pathways and understand the interactions within microbiomes (Jansson, 2013). In contrast to single microbiomes, the connection between microbiomes as well as mutual exchange between them is less understood. Although we live in a highly interconnected world, up to the present date, there are only a few examples of synergistic microbiomes, which have shown that there are important relationships between single microbiomes. The three presented here are the rhizosphere (root–soil connection), the gut microbiome (food–human connection) and the indoor microbiome (plant–inhabitant connection). First, the rhizosphere is one well-investigated example; the root–soil interface is influenced by the plant metabolism via root exudates (Philippot *et al*., 2013). After assessing many experimental data and a lengthy discussion, it was accepted that both the plant as well as the soil influence the composition of the rhizosphere community (Berg and Smalla, 2009). The extent of impact depends on the plant species/genotype, its metabolites and the soil quality. While the rhizosphere is an example of particular importance for plant health, another interesting example which is important for human health is the gut microbiome. David and colleagues (2014) recently provided evidence for the survival of food-borne microbes (both animal and plant-based diet) after transit through the digestive system, and that food-borne strains may have been metabolically active in the gut. Microbial diversity in our gut ecosystem has an enormous impact on the host and vice versa connected by gut–brain cross-talk, which was revealed as a complex, bidirectional communication system (Mayer, 2011). Interesting relationships were detected recently, such as those between the gut microbiome and the development of obesity, cardiovascular disease and metabolic syndromes (Blaser *et al*., 2013) and also on motivation and higher cognitive functions, including intuitive decision making (Mayer, 2011). However, less is known about the food microbiome, although in many countries food is monitored for the occurrence of pathogens while beneficials are often ignored. First studies show that the vegetable and fruit microbiome is highly diverse, and *Enterobacteriaceae* play a substantial role within the vegetable microbiome (Leff and Fierer, 2013; Berg *et al*., 2014). Hanski and colleagues (2012) found a correlation between environmental biodiversity, human microbiota, especially *Enterobacteriaceae*, and allergy, and showed an experimental correlation between bacterial diversity and atopy as shown through significant interactions with enteric bacteria. The current focus of research is placed on the impact of our diet on the composition of the gut microbiome (of particular importance); however, the microbiome in/on our diet opens many more potential insights into the complex interactions. A third example is the indoor microbiome, which has enjoyed enormous attention during the last years due to the fact that we spend most of our time in built environments. The indoor microbiome is also a mixture of variably fixed niches such as bathtubs, kitchen sinks, furniture etc., as well as more moving carriers of microbiomes including the inhabitants (humans and pets), outside air and also of plants (indoor plants as well as outside vegetation). Although we know who contributes to the composition of the indoor microbiome, we don't know how those influences affect the function of the microbial ecosystem. The indoor microbiome is also strongly shaped by cleaning procedures. Can indoor plants and their microbiome positively contribute to human health? Is there an optimal rate of microbial turnover and exchange? All these questions can be answered by studying the shared fractions of microbiome or ‘microbiome connections’. In the three microbiomes presented above, organisms of all domains of life, archaea, bacteria and eukaryotic microorganisms interact to fulfill ecosystem functions. Most of the published studies so far focused only on one microbial group, and therefore it is necessary to make a short note on the interplay between microorganisms from different kingdoms. Recent research has shown that there is a lot of interaction between members of the different groups; e.g. bacteria and fungi interact in the broad range between symbiosis and antagonism (Frey-Klett *et al*., 2011). Little is known about environmental archaea. Interestingly, they are everywhere – from human skin to the endosphere of plants – but often in a low proportion, and their function and interaction with other microorganisms is mainly unknown. Omics technologies can now assist in determining the types of interactions among these organismal groups, ideally by combination of different approaches.

The control of microbial growth is an important area of microbiology, which resulted in significant advances in agriculture, medicine and food science. Biological control is an environmentally sound and effective means of reducing pathogens and pests and their symptoms through the use of natural antagonists or enemies. While in the past mainly single organisms were used, often correlated with inconsistent effects, it is now possible to develop predictable microbiome-based biocontrol strategies (Berg *et al*., 2009). In many cases, diseases are associated with microbiome imbalances (dysbiosis) or shifts, which make it promising to control the whole microbiome. I predict that analysing microbiome connections as well as the microbial interplay opens new doors for advanced biocontrol technologies (ABT). Moreover, ABTs can not only be used to suppress pathogens, they can also be effectively used to establish microbiomes in a desirable, beneficial composition. It should be possible to develop ‘microbiome design’ strategies for particular purposes in the future. Due to the impact of the microbiome on health, growth, size, height, weight, reproduction as well as development of their host, microbiome controls are an attractive goal. Two principles should be considered for the development of ABTs: (i) microbial diversity is an important factor determining the invasion of pathogens (Van Elsas *et al*., 2012), and (ii) synthetic ecology can support the selection of microbes (Dunham, 2007). In all three examples mentioned, biocontrol approaches are applied. Plant health has been the target of biological control for more than 100 years, and now safe and predictable control strategies are making the development of next generation biocontrol products possible (Berg *et al*., 2009). Since we identified the origin and function of rhizosphere microorganisms, indigenous endophyte consortia are showing promise as effective biological control agents. Moreover, in metagenomic approaches to ancient plant-associated microbiomes such as *Sphagnum* mosses, stress protection is identified as the main function (Bragina *et al*., 2014). This could be a valuable response against climate change. In comparison, biological control of the gut microbiome is a new but extremely promising development that has been supported by the enormous success of fecal transplantations (De Vrieze, 2013). Many more applications are, however, possible, e.g. beneficial food microbiomes promoting plant and human health. Further investigation into the impact of the vegetable microbiome on our health seems to be especially important and needs more attention in the future (Berg *et al*., 2014). While functional food can sustain human health, targeted microbial treatment of liquid diets could be used as an additional therapy in hospitals. Last but not least, the indoor microbiome needs our attention and also biological solutions for control. Currently, especially in hospitals and clean rooms, the microbiome is chemically and UV treated, allowing only resistant microorganisms to survive. Additionally, hospital-acquired infections are permanently increasing and are especially caused by (multi)resistant pathogens. In 2014, the World Health Organization produced a global map of antimicrobial resistance and issued a warning that a ‘post-antibiotic’ world could soon become a reality. Woolhouse and Farrar (2014) realized that this phase has already started. They have also emphatically called for a dedicated, coordinated plan of action investigating the root causes of resistance, e.g. the misuse of antimicrobials especially in agriculture, and the development of new drugs and alternative therapies. In those areas, ABTs can make significant contributions to the development of new sanitary measurements and alternative therapies.

Looking even further ahead, I see the continued development and implementation of ABTs and advanced biocontrol products like pro-, pre- or synbiotics containing synergistic microbial consortia or those which induce specific beneficial microbiomes that will contribute to the maintenance and enhancement of microbial diversity for plant and human health and for our environment. The idea to restore the ‘missing microbes’ (Blaser, 2014) is not only important for humans and should be extended to our environment.
